# The economics of antimicrobial resistance in veterinary medicine: Optimizing societal benefits through mesoeconomic approaches from public and private perspectives

**DOI:** 10.1016/j.onehlt.2020.100145

**Published:** 2020-06-05

**Authors:** Didier Raboisson, Ahmed Ferchiou, Pierre Sans, Guillaume Lhermie, Marie Dervillé

**Affiliations:** a IHAP, Université de Toulouse, INRA, ENVT, Toulouse, France; b UR 1303 ALISS, INRA, Ivry-sur-Seine 94205, France; c Université de Toulouse, LEREPS, ENSFEA, IEP de Toulouse, France

**Keywords:** Antimicrobial resistance, Economics, Veterinary, Food animal production, AMs, Antimicrobials, AMU, Antimicrobial use, AMR, Antimicrobial resistance, AMU-H, AMU in human medicine, AMR-H, AMR in human medicine, AMU-V, AMU in veterinary medicine, AMR-V, AMR in veterinary medicine

## Abstract

Antimicrobial resistance (AMR) is a global public health threat driven by a combination of factors, including antimicrobial use (AMU) and interactions among microorganisms, people, animals and the environment. The emergence and spread of AMR in veterinary medicine (AMR-V) arising from AMU in veterinary medicine (AMU-V) can be linked to individuals' economic behaviour and institutional context. We highlight the limitations of current microeconomic approaches and propose a mesoeconomic conceptual model of AMR-V that integrates actors' strategic and routine behaviours in their context from a dynamic perspective using the concepts of externality, globality and futurity. The global solution to AMR-V management relies on a trade-off between i) the global externality assessment of AMU-V with respect to AMR-V (public perspective) and ii) farm- or value chain-level marginal abatement cost evaluation (private perspective). The improvements realized by the proposed mesoeconomic conceptual model include i) the simultaneous fight against the emergence and spread of AMR-V and ii) a local decrease in AMU-V without any loss of competitiveness for private actors due to the development of adequate production standards. A set of generic equations describing the stepwise change in the scale of analysis is finally proposed. This original contribution to the global challenge of AMR through a mesoeconomic approach bring substantial improvement for better AMU. This model can be considered a way to smoothly promote institutional change and a call for public policies that support public private partnership in the development of adequate incentives. The model requires further development prior to its application in a given value-chain or territory.

## 1 Introduction

Antimicrobial use (AMU) in veterinary medicine (AMU-V) plays a crucial role in improving animal health and welfare. Antimicrobials (AMs) are used in food animal production for the curative treatment of diseased animals, for metaphylactic treatment (the group treatment of healthy and infected animals) to control the spread of diseases, and for the prophylactic treatment of healthy animals to prevent infections in cases of high risk for diseases. In some countries, AMs are still used at sub-therapeutic doses to improve growth performance. In food animal production, AMU decreases farms' economic vulnerability due to the risk for diseases and is also related to public health through its potential to control zoonotic disease. Despite the medical and economic significance of interests in AMU, it is recognized that AMU-V can result in the side effect of antimicrobial resistance (AMR), which can subsequently be transmitted to humans, usually through contact with infected animals, the environment and the food supply chain [[1], [2], [3], [4]]. AMR refers to the ability of microorganisms (bacteria, viruses, parasites and fungi) to withstand the effect of AM agents, resulting in the decreased effectiveness of AMs in treating infectious diseases. AMR in veterinary medicine (AMR-V) can lead to treatment failure with direct negative effects on animal health, welfare and productivity and on farms' economic success [5]. In the public health context, additional costs associated with AMR are incurred due to more expensive AMs, longer hospitalization time, longer sick leave, a higher mortality rate and more research and development (R&D).

AMR has reached increasing and alarming levels, which appear to be tightly related to the overuse and misuse of antibiotics [[6], [7], [8]]. Currently, there is a consensus regarding the positive associations between AMR and AMU [7] despite the lack of the clear quantification of this process and the differences observed across classes of drugs. Once created, AMR is not eradicable. Although AMR can decrease when AMU decreases, AMR genes can move between bacteria, hosts and environments. The usual solution, that is, the development of new biotechnological solutions (new AMs), remains very complex and costly [9], which has led to the activation of social, economic and institutional levers in both the human and animal sectors as an alternative to the very low likelihood of success of the solutions provided by biotechnology. Decreasing AMU, specifically focusing on the overuse and misuse of AMs, is then often seen as the key positive action in the short and long term for both the human and animal sectors. For instance, since 2002, a French campaign called “antibiotics are not automatic” has been developed to decrease human AMU. In 2001, the European Union (EU) banned the use of antibiotics for growth-promotion purposes. In December 2013, the US Food and Drug Administration recommended that farmers voluntarily phase out the use of antibiotics as growth promoters [5]. In addition, several high-income countries have implemented measures to reduce AMU-V [10]. In 2012, France implemented an EcoAntibio plan, with the aim of reducing the use of veterinary antibiotics by 25%. A 37% decrease in AMU-V over a 5-year period was achieved, suggesting that alternative practices to AMU-V for the management of animal health and welfare exist and that stakeholders are willing to implement these practices. This altogether shows that there is no major technical or organizational lock-in with respect to AMU-V, at least in short term.

Extensive literature-based sociological, psychological and microeconomic approaches (centred on individuals and farms) have attempted to shed light on the drivers of behavioural change in terms of AMU, leading to a clear overview (section 2) of the determinants of AMU in food animal production. These approaches are useful support the decrease in AMU-V, but they do not shed light on the collective optimal AMU-V, which should consider trade-off between individual and collective interest and include several criteria to integrate all the functions that animals provide to society [11]. The findings concerning the economics of AMU in human medicine (AMU-H) based on the concepts of externality, globality and futurity [12] clearly show that mesoeconomics and macroeconomics are complementary in analyses of AMU-V. Macroeconomics considers economy as a whole, aims to identify the drivers of growth or income and could shed light on trade-offs at the national or supranational level. Therefore, macroeconomics is required for monitoring global issues, such as AMR. However, a good understanding of the mesoeconomic drivers of change is also required. Mesoeconomics refers to an intermediary approach between microeconomics and macroeconomics that clearly considers collective action and interactions between market institutions and individual choices. Coordination devices, such as contracts and quality conventions, that render individual behaviours compatible and support value creation and sharing are considered. Therefore, mesoeconomics overcomes the usual limitations of microeconomics approaches to AMU and considers externality and globality. Such a demonstration is provided in section 3, which shows that a regional AMU decrease without any loss of competitiveness in the food animal production sector is the optimal solution for society. The practical application of these concepts (section 4), from microeconomics to mesoeconomics, demonstrates that optimizing the net benefits of AMU-V considering the scope of coordination allows immediate regional win-win solutions.

## 2 Understanding the microeconomic determinants of AMU in food animal production to help decrease AMU

There is widespread agreement that AMR and AMU are strongly influenced by actors' economic behaviour and institutional context [13]. By studying individual decision-making processes, microeconomics can help with understanding farmers' behaviour in disease management and in developing innovative responses to AMR-V. The dual situation of veterinary drugs has been highlighted in a recent framework [14]: AMs are simultaneously drugs, and thus are regulated in many countries, and production factors, and thus substitutable for other production factors, depending on farmers' behaviour and choices. The farmer is the cornerstone of AMU at 2 levels: first, he/she decides to treat or not treat a diseased animal with AMs, and second, he/she influences farms' AM demands by managing disease risk factors. As patients in human medicine, farmers play a role through AM-induced demand, even if the drug is regulated and prescribed by veterinarians. The potential determinants of AMU can then be derived from three groups of drivers: (i) farmers' characteristics (age, sex, ability to detect disease, expertise, risk aversion, time preference, etc.), (ii) farms' structural characteristics, related practices and risk of disease onset, and iii) the economic and institutional conditions under which farms operate or generate revenue.

First, the risk of disease onset and farmers' preferences and characteristics are key determinants of AMU [14,15]. Before farmers use preventive treatment (AMs or other treatments), they evaluate (i) the risk of disease onset, (ii) the potential economic impact of the disease if it occurs, and (iii) the effectiveness of curative treatment with respect to disease occurrence. Such evaluations and consequent AMU depend on farmers' characteristics and preferences. On the one hand, AMs can be seen as a risk-decreasing input, and a risk-averse farmer will tend to overuse AMs to prevent disease. A risk-averse farmer always seeks to evaluate potential risks to prevent them, but such evaluations mainly rely on farmers' technical skills (ability to detect disease) and knowledge. On the other hand, the substitutions between prevention and curative treatment, and mostly between the medical and non-medical practices used to manage diseases, represent the key basis of the farmer's position as a moderator of AM demand. These substitutions also introduce the issues of farmers' time preference in 2 main ways. First, a different time scope should be considered since prevention should be undertaken several months or even years before potential disease occurrence and may require specific training, and time preference prioritizes (late) curative treatment over (early) prevention. Second, the risk of disease occurrence remains in the case of prevention, sometimes leading to both prevention and curative treatment and weakening the risk-aversion principles that favour prevention.

Second, farms' characteristics represent the structural parameters (livestock buildings, farm size, labour force, etc.) that influence practices (hygienic conditions, extensive production systems, biosecurity measures, etc.) and disease onset since such practices are the result of the structures and constraints of farms and the strategies of farmers [16]. These practices can change and be adapted through simultaneous technical changes [15]. In the short term, these practices act as constraints for farmers and can be modified from a long-term perspective.

Third, there is a consensus that institutional conditions are key determinants of AMU, and such conditions are accounted for in microeconomic studies as exogenous constraints. These conditions include farm input and product prices, farming process regulations, and consumer preferences and, more specifically, AM prices, markets and access. The low prices of AMs relative to their expected efficacy make price an important factor in AMU [6], which has been demonstrated in studies describing the arrival on the market of generic veterinary AMs, which have been associated with reduced prices for farmers and an increased use of this class of AMs [17]. We can extrapolate that an increase in the price of AMs may be associated with a decrease in AMU, provided that alternatives to AMs exist. However, the relationship between AM prices and AMU highly depends on institutional factors, and predicting that an increase in AM prices will result in a decrease in AMU is speculative. AMU depends not only on AM price elasticity but also on local regulations and routines, famers' private contract constraints, veterinary prescriptions, pharmaceutical firm marketing strategies, etc. For instance, in the French bovine context, the introduction of new AMs in the cattle production market was not associated with an increase in AMU [18]. In countries where AMs are mainly delivered by veterinarians, the risk of AM overuse arises from veterinarian behaviour since a large part of the revenue of veterinarians depends on the sale of AMs [18]. This risk of overuse has led to a call for decoupling veterinarians' right to both prescribe and deliver AMs. AMU policy may also be used as a commercial argument, non-tariff barrier or way to reinforce non-cost competitiveness. Conversely, AMR may alter the reputation of a value chain.

**Fig. 1:**
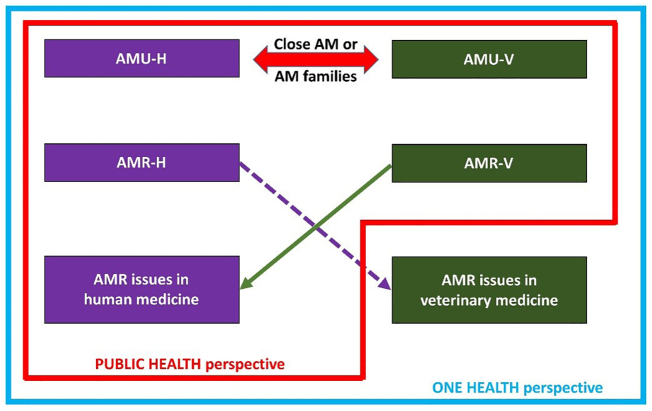
Antimicrobial use and antimicrobial resistance : public health and one health perspectives

Two main developments are required in regard to the present state of the art on the economics of AMU-V. First, the abovementioned literature focuses on veterinary drugs (AMU-V). Consequently, the situations in human and veterinary medicine cannot be investigated independently (Fig. 1). A “one health” approach highlights that AMU-V and AMR issues in humans are intrinsically linked, in that AMR issues in humans are the sum of AMR in human medicine (AMR-H) (arising from AMU-H) and AMR-V (arising from AMU-V). Both uses of AMs partially use the same classes of molecules, and thus, AMU-V can have consequences for public health. These relations are the basis of the externality issue described below. Similarly, AMR issues in veterinary medicine are the sum of AMR-V (arising from AMU-V) and AMR-H (arising from AMU-H). This situation remains to be analysed and represents dramatically lower stakes compared to the opposite relationship (Lhermie, 2020). Second, the extensive microeconomic literature on AMU-V poorly accounts for the collective and regional levels, calling for mesoeconomic approaches. For instance, institutions are accounted for in the microeconomics of AMU-V as determinants of behaviours, but they are considered static and external [14]. Region-level efforts to account for the collective capacity of innovation and reputation building are lacking. We propose to extend the economics of AMU-H to AMU-V to address the 2 limitations mentioned above.

## 3 From a micro- to mesoeconomic conceptualization of AMR-V (arising from AMU-V)

As in human health [12,19], three key concepts (externality, futurity and globality) are proposed here to examine how economics can help in developing innovative responses to AMR-V. These three key concepts highlight the needs and potentialities of the mesoeconomic approach.

### 3.1 Externality

An externality is a cost or a benefit that affects an actor or group of actors who did not choose it. An externality is a market failure because a party is affected by a transaction without receiving financial compensation [19]. Externality is related to the joint production of goods or services in which one good or service is not tradable. AMR is conceptualized in economics as a negative externality resulting from the use of AMs. AMR has an undesirable effect on people other than the immediate users of AMs and is consequently associated with the costs that are incurred, without any compensation, by people other than the agents who are directly involved in AMU. Market failure is linked to the producers, prescribers, and users of AMs, who do not pay the costs of AMR incurred by society. Two kinds of externalities due to AMU-V can be defined. In a “one health” approach (Fig. 1), the impacts of AMR-V on both veterinary and human medicine should be considered since decreased AM efficiency is a long-term threat to the farm animal sector. The farm animal sector plays an important role in food security (and capital) in many countries. The moral and ethical stakes involved in AMR-V are dramatically higher for human medicine than for veterinary medicine [20]. From a public health approach, the intra-animal sector externality due to AMU-V (i.e., the effect of AMR-V on veterinary medicine only) is seen as a private issue at the value chain level (even if there are externalities between actors within this sector).

Attempts to internalize the externality of AMU-V for human health have emerged in some countries through the implementation of taxes on AMU-V. For example, since 2014, in Belgium, every distributor of veterinary antibiotics with market authorization must pay a tax [21]. However, the amount of the tax has generally been adopted in an arbitrary manner without any economic evaluation of the abovementioned externality. In addition, the externality of AMU-V for human medicine remains difficult to quantify due to the imprecise quantification of the impact of AMU-V on AMR in human health [22]. The link between AMU and AMR transmission and the role of the environment in the spreading of AMR have been shown to greatly differ between low- and high-income countries [23]. This kind of tax is a political initiative that aims to change the behaviour of individuals, which may substantially change the level of AMU. However, the implementation of taxes at the national level in an open economy and in the absence of product differentiation may fragilize the national industry, without social benefits at either the national level or the local level (AMR will arrive from outside in the case of product substitution). Taxes should be Pigouvian since they are neutral with respect to the regional industry and independent of the issue of the emergence and spread of AMR from outside the area of the tax application (the globality issue of AMR). Pigouvian taxes are especially designed to correct market failure due to externalities. In the present situation, the tax may increase the marginal private cost of AM up to the amount of its negative externality.

### 3.2 Globality

The global nature of the issue of AMR refers to its rapid geographical (worldwide) spread, suggesting coordinated control policies (similar to those for other global issues such as climate change). AMR is recognized as a global public health issue [9,10] since it currently concerns all regions of the world, and the emergence of new resistance is followed by a quick spread of AMR worldwide due to the current global increase in the circulation of people, animals and products. The global nature of AMR concerns both AMR-H and AMR-V and has led to the definition of two strategies to fight it: the fight against AMR emergence and the fight against AMR spread. This mainly means limiting the emergence and the spread of AMR-V to humans. For AMR-H, the global nature of AMR suggests that fighting against the emergence of AMR-V holds little social significance if no action to limit the spread of AMR-V is taken. The key feature arising from globality for local decision makers around the issue of AMR is as follows: from a local perspective, efforts to contain the emergence of AMR-V may offer limited benefits if nothing is done to prevent the spread of AMR-V that emerges elsewhere. This feature is the first main lesson of the mesoeconomic approach to AMU-V: market functioning – the circulation of animals and products – must be considered to analyse issues related to AMU-V. For a given country, fighting against the emergence of AMR-V without containing the spread of AMR-V does not make sense and does not fit the optimal socioeconomic strategy. Most of the local initiatives (national or regional programmes) adopted to date have focused on AMR-V emergence and underestimated the importance of AMR-V spread. For instance, in France, AMU-V decreased by 37% from 2012 to 2017, and EU policies were implemented to ban or limit the use of certain AM classes such as critically important antimicrobials (CIAs) in veterinary medicine [6,24]. However, no simultaneous measures were taken to prevent the import of animals and products as goods originating in countries without these constraints. To deal with the global nature of AMR-V or AMR-H, the optimal solution may rely on the global regulation of AMU. Global initiatives are now in progress, for instance, under the aegis of the WHO or OIE [10,25], but most of the current actions remain country or region centred; for example, in the EU, there are many national plans, but to date, there has been no EU-level coordination. International coordination will take time and use examples of other global efforts, such as carbon regulations or COP 21 initiatives, highlighting the complexity of global coordination issues and the difficulties in reaching a compromise.

The global characteristics of AMR, however, reduce the expected societal benefits of local initiatives, reducing the emergence and the local spread of AMR. It is quite difficult to know whether the residual societal benefits is positive or null, but this situation calls for local initiatives that do not hamper the competitiveness of the food animal production sector, i.e., that do not increase the cost of production with additional constraints for producers. Local initiatives that threaten the local food industry may lead to local societal losses. Even worse, in case of an increase in the import of products from countries with lower quality standards, AMR may spread anyway. This situation may be observed where national plans that aim to decrease AMU-V are applied. In the Netherlands, for instance, the rate of multi-resistant bacteria is much higher in imported chickens than in chickens produced within the country [26]. This situation leads to the second main lesson of the mesoeconomic approach to AMU-V: an improvement in the overall benefits linked to AMU remains possible under the double condition of reducing the AMU-V in a given geographical area while ensuring that this objective is achieved without any loss of competitiveness. This lesson highlights the need to link AMR with market dynamics. Local measures to contain AMR may be effective if they concern both the emergence and spread of AMR, for instance, by protecting local systems from import of AMR through the development of quality standards, including specifications related to AMU. Product differentiation is a way to create value in the food market; it implies entering or creating new markets [27]. Indeed, the material and immaterial specificity of products may generate an attachment on the part of the consumer and a willingness to pay a higher price. Such specificity requires the collective building of resources to make market differentiation strategies effective and entails reputation building based on an identity and an evaluation mechanism, as in the case of quality signs such as protected geographical indications or organic farming. Indeed, quality is not only material (tangible) but also conventional (intangible) based on a shared and objective representation of goods.

### 3.3 Futurity

The futurity issue arises from the fact that the costs of AMR are spread among current and future generations and the fact that because of the uncertainty of future effects and discounting over a long period of time, future costs are considered negligible in many economic evaluations. The absolute costs of AMR have been discounted on the basis of time preferences, de facto resulting in very small costs [28,29]. This situation leads to the widespread use of AMs rather than the saving of AMs for future use. To circumvent these issues, the proposed solutions include the choice of null discounting rates [28,30]. Uncertainty regarding the future evolution of AMR has yet to encourage the *status quo* in AMU, with optimist expectations of a lower average impact of AMR given new R&D. There is now a consensus that the development of new AMs is unlikely and may scarcely occur, reinforcing the need for transparent and accurate AMR cost assessment. In the context of food animal production, assessing the externality of AMU-V for human medicine will require including a null discounting rate to obtain the accurate value of AMU-V externality internalization. In contrast, an “usual” discount rate is required to calculate the net present marginal cost of a decrease in AMU-V since this assessment is performed in a private sector-like context. Similarly, the usual discount rate is required when analysing the externality of AMU-V for veterinary medicine (private sector issue).

The under-evaluated impact of AMR on society has resulted in a lack of incentives for developing appropriate strategies to control the emergence and to reduce the spread of AMR [28,31]. Futurity, in the sense of time preference, suggests the preference for short-term incitation rather than long-term regulation. However, futurity, from a mesoeconomic perspective, is also a way to build common projects for the future and to overcome short-term conflicts [32,33]. The elaboration of a standard for the reasonable use of AMs and the engagement through contracts for processors and retailers to market such products correspond to the building of a common future. The setting of such collective rules and their enforcement are a way to change individuals' ways of thinking and behaving [34,35]. Futurity, in the sense of building a common (mesoeconomic) future, is a way to support institutional change and, therefore, the efficiency of public incentives that aim to reduce and optimize AMU.

In summary, the present contribution to the conceptualization of AMR-V leads to the following main approaches: a global externality assessment of AMU-V from the public perspective (the human health of future generations) and a farm- or value chain-level marginal abatement cost evaluation of a decrease in AMU-V from a private perspective (the competitiveness of the animal industry). These represent the 2 main expected approaches to AMU-V in the future. Potential global solutions that address these 2 perspectives with opposite interests require an international agreement to be efficient. The mesoeconomic perspective enables the search for pragmatic solutions and trade-offs that are adapted to the local risk of the emergence and spread of AMR-V. The last section highlights how these 3 economic concepts may be practically applied from microeconomics to mesoeconomics in an attempt to define the win-win strategic trade-offs that policy makers may seek.

The economic evaluation of AMU-V reduction can also contribute to the elaboration of a trade-off between economic and social outcomes and prevent a loss of competitiveness.

## 4 Mesoeconomic approach to AMU-V to decrease AMR: How animal health actors may coordinate

Here, we attempt to see how a trade-off between public global externality assessment and private marginal abatement cost evaluation may be achieved by considering different scales of analysis to define win-win strategies. The reasoning will start with the microeconomic perspective of farmers and will then extend to the mesoeconomic perspective of the region. For ease of reading, the associated benefits, extra costs and negative externalities are written in green, blue and red, respectively.

Let us first consider the benefits resulting from AMU at the farm level. The net benefits ${\mathrm{NB}}_{\mathrm{Farm},d,t}^{\mathrm{AM}}$ for a farm, given the quantity of AMs used to treat animals for a given disease *d* at time *t,* can be expressed as follows:(1)
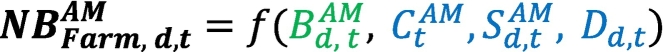
where  denotes the benefits (avoided losses) associated with AMU to treat disease *d* at time *t*;  denotes the costs of AMs, including their administration costs to animals at time *t*;  denotes the farm-level costs associated with the undesirable side effects resulting from AMU to treat disease *d* at time *t*; and  denotes the diagnostic costs of disease *d* at time *t*. The side effects  mainly refer to AMR created within the farm that will decrease the expected efficacy of future AMU in the farm. Because  remains within the farm, it is not considered an externality. The direct benefits for the farmer, , represent the difference between the production obtained with AMs and that without AMs; it includes not only benefits for the treated animals for disease *d* but also benefits for any other subclinical infections that AMs are allowed to treat at time *t*, as well as benefits for other animals of the batch at time *t* through the lowering of the risk of infection transmission to congeners. The latter two benefits are not externalities, as the beneficiary is the same actor (the farmer). To include the positive and negative externalities resulting from AMU, Eq. (1) was extrapolated at the regional level (for the production sector only) in Eq. (2). The positive externality is related to the reduction in the transmission of infectious diseases among neighbouring farms, given AMU. In contrast, the negative externality is related to the emergence of and increase in AMR due to AMU.(2)
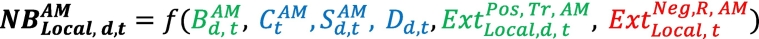
where  denotes the local positive externality (i.e., reduction in disease transmission *Tr* to neighbouring farms) associated with AMU for disease *d* at time *t*, and  denotes the local negative externality (i.e., emergence or increase in AMR) associated with AMU at time *t*. The local negative externality  refers to whole AMR due to AMU-V, i.e., AMR issues in human and veterinary medicine (Fig. 1). Thus,  refers to resistance to AM for i) livestock in the surrounding areas and ii) the whole human population, including (iii) farmers and their families. The term  can easily be divided into 3 sub-terms to match this externality of livestock, human population and farmer families. This categorization is not applied here to help maintain the following equations as simple as possible.

Although farmers are at the forefront of AMU decisions through their farm management practices, firms (slaughterhouses, food-processing companies or retailers, and consumers) that buy animals (or animal products) can influence AMU routines by requiring low-AMU production standards. The development of such standards involves the consideration of both the intangible and tangible characteristics of the good. Low-AMU products could indeed be less homogeneous than standard industrial products since the high prevalence of disease leads to heterogeneous products due to change in growth and metabolism. Requiring homogeneous products (standardized weight, colour, etc.) has indeed been proven to be a factor involved in AMU on the farm [13]. Any reduced AMU involves a decrease in the negative externalities created by AMU on the food-processing companies, including a reduction in (destruction of) companies' reputation among consumers and other collateral damage. For instance, in France, in the case of antibiotic residues in a tank of milk, the product is destroyed, and the non-compliant farmer is penalized; however, the dairy company is also indirectly penalized via, for example, a disruption in its procurement plan. The net benefits for a food-processing firm can be defined as follows:(3)
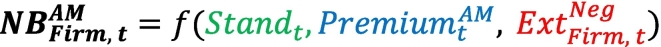
where ${\mathrm{NB}}_{\mathrm{Firm},t}^{\mathrm{AM}}$ denotes the net benefits for a firm that imposes production quality standards with respect to AMU to buy animals or animal products from farmers;  denotes the level of production quality standards, i.e., tangible or intangible quality, defined by the firm for buying animals (or animal products) at time *t*;  denotes a premium provided to the farmer with respect to good AMU standards; and  denotes the negative externalities for the firm given the use of AMs by farmers at time *t*. The term  may be divided into tangible or intangible qualities, but such categorization is not applied here to maintain simple equations. The negative externalities  can be compensated through new production standards  associated with a premium  for farmers. These relationships are further described through the definition of several regimes of AMU (or regimes of product quality) as indicated in Table 1.

**Table 1:** Regimes of the use of AMs based on the scale of the sector.

Regimes of AMU	${\mathrm{Premium}}_{t}^{\mathrm{AM}}$	*Stand*_*t*_	${\mathrm{Ext}}_{\mathrm{Firm},t}^{\mathrm{Neg}}$
Conventional regime	= 0	Conventional quality	Reduction in company reputation, disruption in the procurement plan
Organic agriculture (OA)	> 0	OA quality; high AMU-related intangible quality	Reduced or eliminated
Sustainable with premium	> 0	Fixed tangible quality standard; high AMU-related intangible quality	Reduced or eliminated
Sustainable with flexibility	= 0	Variable tangible quality standard; high AMU-related intangible quality	Reduced or eliminated

The conventional regime represents a starting point with the absence of a premium paid by the firm and the presence of negative externalities. The market with a premium can be reached by the farmer through farming technical solutions allowing for low AMU. For example, the organic agriculture quality regime allows for a reduction in such externalities given the organic standards of production set by the firm and the premium that it offers for the quality of the products. Other trade-offs (e.g., sustainable regimes) can be suggested by reducing externalities by setting a desired level of production quality standards and defining a premium for compensating farmers' efforts to comply with the standard. A more innovative (or flexible) regime aims to allow for changes in the product standard and to define a premium corresponding to the product standard. A reduction in the strict product characteristics that the industry (firms) requires of farmers is a key driver for a decrease in AMU since heterogeneity in the size or weight of animal products is severely penalized by the agriculture industry. The extra cost that the firm will face due to product heterogeneity upon arrival of the product at the slaughterhouse or manufacturing facility, in the present context of high food product standardization, may be substituted with the  that firms have to face.

By combining Eqs. (2), (3), in Eq. (4), we define the net benefit *NB*_*Chain*, *d*_^*AM*^at the value chain level (production and food processing) resulting from AMU to treat animals for disease *d* at time *t.* The collective benefits of AMU  (Eq. (3)) are divided into the following 3 items (first line of Eq. (4)): a benefit linked to disease control (considered a control of potential production damage ), the change in the firm's production standards , and the premium  perceived by the farmer and paid by the firm.(4)
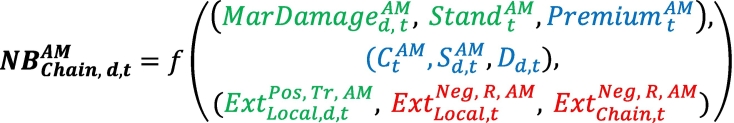
where  denotes the marginal production damage controlled by AMU, and  are the negative externalities supported by the entire value chain (including the industrial firm). Dividing  into  and  allows us to represent the biologic point of view (how AM may control disease) and the value chain point of view (product obtained), respectively, and provides an overview of the trade-off surrounding AMU. Both remain benefits (i.e., green) when considered together at the value-chain level.

Because veterinarians play a key role in AMU in many countries, as the exclusive prescribers and main sellers of veterinary drugs, the net benefits of a veterinary structure can be defined as a function of advice and prescription activities, on the one hand, and of the delivery of veterinary drugs, on the other hand:

(5) $${\mathrm{NB}}_{V\mathrm{et},t}=f\left(Q_{t}^{\mathrm{AM}}\times {\mathrm{Marg}}_{t}^{\mathrm{AM}},{\mathrm{Adv}}_{t}^{\mathrm{Vet}}\right)$$

where *Q*_*t*_^*AM*^ denotes the quantity of AMs sold by the veterinary structure (bought by the farmer); *Marg*_*t*_^*AM*^ denotes the margin of the veterinary structure for AMs delivered (sold); and *Adv*_*t*_^*Vet*^ denotes the income of the veterinary structure associated with advice and prescriptions provided to farmers at time *t*.

By combining Eqs. (4) and (5), the net benefits *NB*_*Chain*, *d*, *t*_^*AM*^ for the value chain (currently defined as farms, food-processing firms and veterinary structures) resulting in AMU to treat animals for disease *d* at time *t* can be expressed as follows:(6)
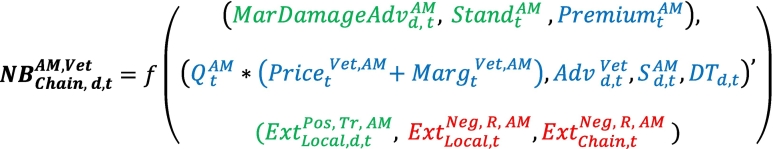
where  denotes the marginal production damage control function in the context of high veterinary advice;  denotes the purchase price of AMs by the veterinary structures; and  is the diagnosis tests (out of diagnosis through veterinary consultation, accounted for in ). The term  in Eq. (5) becomes  in Eq. (6) to reflect that more appropriate AMU is permitted by better and more appropriate veterinary advice (as accounted for in ) as follows: the marginal benefit of controlling damage (disease) by AMU is increased.

Eqs. (1), (2), (4), (6) define the net benefit of AMU at different levels and for different actors. All the equations are simplified since the risk of occurrence of the disease to be treated by AMU is conditioned on the so-called risk factors of diseases (breeding conditions, farmers' routines and practices). The definition of the conditional function makes it possible to integrate the risk of disease, the means of prevention and the temporality of events since prevention is realized and paid for at time t and the possible disease and its treatment are realized and paid for at time t + 1. When applied to Eq. (6), the conditional function is given in the right-hand term of Eq. (7), and its left-hand term is the same as that in Eq. (6) after adjustment for the time lag.(7)
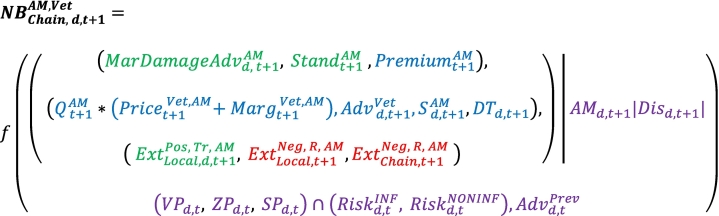
where  denotes the use of AMs to treat disease *d* at time *t* *+* *1*;  denotes disease *d* detected at time *t* *+* *1*;  denotes the costs linked to vaccines for the prevention of disease *d* at time *t*;  denotes the costs linked to new (non-medical) practices to prevent disease *d* at time *t* (i.e., non-medical measures such as hygiene, housing, and feeding);  denotes the costs of the structural prevention of disease *d* at time *t* (i.e., barn characteristics, farm structures including barn characteristics and easiness of work);  denotes the infectious exogenous risk at time *t* for animals of contracting disease *d* at time *t* *+* *1*;  denotes the non-infectious exogenous risk at time *t* for animals of contracting disease *d* at time *t* *+* *1;* and  denotes the income of the adviser (veterinary or other) associated with the preventive advice at time t. Reading Eq. (7) from left to right shows that exogenous risks in interaction with farmer practices (last line) influence disease occurrence (), which, in turn, influences AMU (), which, in turn, influences the benefits, costs and externalities of AMU (first block).

Eq. (7) represents a basis for many potential economic assessments, including i) the farm- or value chain-level marginal abatement cost of  (private perspective), ii) interventions, such as Pigouvian taxes, for changing practices without industrial sector disruption and iii) the global externality assessment of AMU-V with respect to AMR-V (public perspective).

First, the marginal abatement cost of  holds high significance since the decrease in AMU-V to be achieved in national plans is often adopted somewhat arbitrarily, whereas the evaluation of the marginal abatement cost of the decrease in AMU-H has been recommended for the past 2 decades [28]. The assessment of the monetary and non-monetary costs of each unit of an AMU-V decrease because of the new constraints farmers face should be a priority for every new regulation in the animal health sector. The marginal abatement cost of  is accurate if it is defined as the minimum of the solutions provided by a set of substitutions between inputs or outputs in the short and long term in the agricultural production process, including the food animal industry and its supporting sectors, when  is forced to decrease. The marginal abatement cost of  is overestimated in the case of an economic assessment that does not include all the combinations of input/output substitutions permitted by Eq. (7). Second, Eq. (7) helps in testing Pigouvian taxes to reintegrate in the market the negative externalities from  without any loss of competitiveness for private actors by playing with the input/output substitutions instead of levying arbitrary taxes on AMU-V. The typical example of such a Pigouvian tax is the reinforcement of prevention instead of disease treatment through vaccine premiums and AM taxes. Third, the regional or even global solution to AMR-V management relies on the trade-off between i) the externality assessment of AMU-V with respect to AMR-V (public perspective) and ii) farm- or value chain-level marginal abatement cost evaluation (private perspective). Partial or general equilibrium models or agent-based economic models can be easily developed from the present mesoeconomic framework, for instance, in an attempt to define new coordinated control policies (as in the case of climate change issues). The fact that null and usual discounting rates are required for externality and marginal abatement cost evaluations, respectively, may greatly influence the optimal trade-off in the economic evaluation and should be seen as a major criterion that influences the optimal solution.

To contribute to the above findings, Eq. (7) can be developed to consider AMs being used differently for preventive, metaphylactic or curative purposes and to include other actors such as pharmaceutical firms and farm advisors. Eq. (8) triplicates the first part of Eq. (7) into curative AM (AMc) and metaphylactic AM (AMm), both of which are located in the first part of the equation since they are used when diseases are observed (i.e., t + 1), and prophylactic AM (AMp), which is located at the end of Eq. (8) since it is used before the appearance of the disease (i.e., t). This last part behaves similar to other preventive instruments. , , and  from Eq. (7) are written as the sum of different instruments in Eq. (8).  is also considered as the sum of the quantity of vaccines multiplied by the price the veterinarian pays for them and his/her margin.(8)
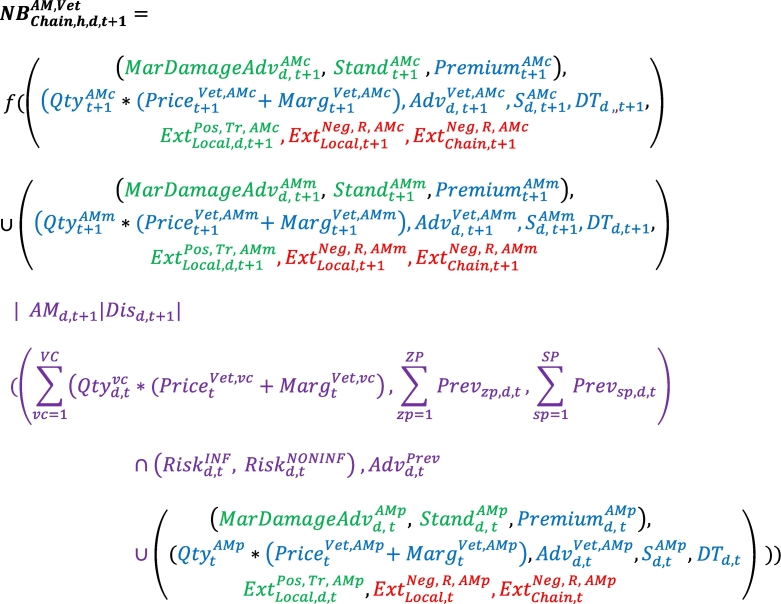


This approach extends the combinations of substitutions among drugs (vaccines vs AMs), between medical and non-medical inputs, between structural investments and daily practices and between services provided to farmers by different advisors (paid services or free services, paid when the product is bought). Importantly, analysing the substitutions taking place at the farm level within a mesoeconomic framework automatically accounts for markets, collective organization and the stakeholder categories involved in farms and their opportunities outside of the agricultural sector (different species for agribusiness firms) and even out of the agricultural domain (pets for veterinarians). Table 2 presents an illustration of the substitutions among different combinations of inputs or outputs. To match the win-win strategies previously described, the revenue of Pigouvian taxes may be allocated to disease prevention support (vaccines) as an application of the principle of lower AMU-V without disruption of the business model (case 1). At the veterinary office level, the substitutions among revenues from drug delivery and advice paid for risk management (herd medicine) are also related to AM stewardship (case 2). The value chain strategy previously described is also considered (case 3). A precision approach based on differentiating antibiotic classes depending on their AMR consequences (e.g., digestive flora exposition) through pharmacodynamics and pharmacokinetic characteristics is also proposed (case 4) through substitutions between AM externalities and the marginal control of the damage that farmers confront.

**Table 2:**
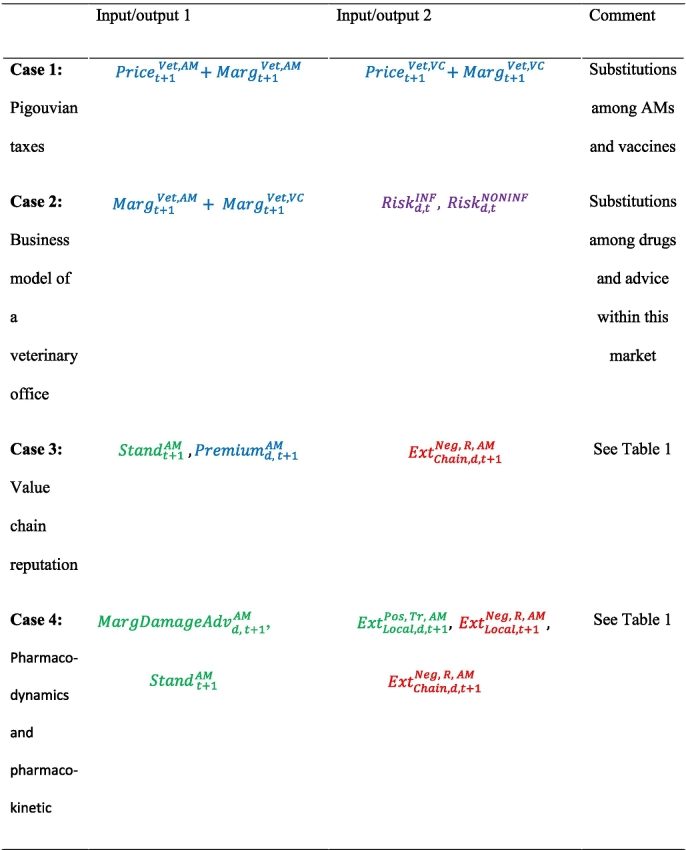
Example substitutions between different sets of input/output combinations.

## 5 Discussion

The present work represents an original contribution to the global challenge of AMR. The main aim was to demonstrate the usefulness of a mesoeconomic approach. The present work shows that this level of analysis may support substantial improvement in better AMU. This work shows the ability to develop new local solutions by better coordination among value-chain actors. A decrease in AMU is permitted by a win-win strategy that gathers and accounts for private interests and public outcomes. The (non-exhaustive) list of potential substitutions (Table 2) clearly shows the possibility of the practical implementation of such a strategy. The recent emergence of “antimicrobial-free” value-chains demonstrates the relevance of the AMU regime described in Table 2. This example supports the present demonstration that decreasing AMU while maintaining sector profitability is achievable by providing better coordination among the actors within the value chain supporting value creation and fair distribution.

The present approach achieves a better societal benefit by improving AMU without any shock within the animal production sector. The consumer point of view is not directly included in the model but is indirectly considered through the definition of new standards (Table 2). These standards are closely linked to consumer demand and willingness to pay. The proposed approach can be considered a way to smoothly promote institutional change while avoiding shock and social conflict. Such a positive transition requires public policies that support public private partnership in the development of adequate incentives.

The present work proposes a generic approach to the question of AMU in the animal production sector and requires further development prior to its application in a given value-chain or territory. The correct parameterization of such model equations may require several datasets that are often difficult to find simultaneously in the same area and sector. Defining the biological and economic substitutions of practices and routines that help in AM better use (Table 2) could also require assumptions regarding actor behaviours from private companies to individual famers.

## 6 Conclusions

The comprehensive mesoeconomic approach to AMR-V proposed here includes an economic conceptualization of AMR-V gathering private and public perspectives, from a public health or a “one health” perspective, that permits the definition and assessment of strategies to contain AMR-V without a loss of competitiveness for the food animal production sector. The present work contributes to the conceptualization of the AMR-V issue by precisely defining two approaches to AMU-V that are expected to become popular in this domain in the future, namely, a global externality assessment of AMU-V from a public perspective (human health) and a farm- or value chain-level marginal abatement cost evaluation of an AMU-V decrease from a private perspective (intra-animal sector). The present work demonstrated the usefulness of moving from the microeconomic approach to the mesoeconomic approach to AMU-V. Further development of macroeconomic approaches to AMU-V is needed to better address the issue of globality related to AMU.

## Funding

This research did not receive any specific grant from funding agencies in the public, commercial, or not-for-profit sectors.

## Role of the funding source

Not applicable.

## Informed consent

Not applicable.

## Animal welfare

Not applicable.

## Availability of materials

Not applicable.

## Declaration of Competing Interest

We hereby certify that the none of the authors have any conflict of interest for the present manuscript.
